# 4532 Pancreatic Cyst Risk Stratification for Early Detection of Pancreatic Cancer Using Quantitative Radiomics and Activity-Based Biomarkers

**DOI:** 10.1017/cts.2020.417

**Published:** 2020-07-29

**Authors:** Sophia Hernandez, Andre Luiz Lourenco, Evan Calabrese, Tyler York, Alexa Glencer, Spencer Behr, Zhen Jane Wang, Eugene Koay, Charles Craik, Kimberly Kirkwood

**Affiliations:** 1 University Of California, San Francisco

## Abstract

OBJECTIVES/GOALS: Pancreatic cysts are comprised of both precancerous mucinous lesions and non-mucinous lesions with minimal malignant potential. Our goal is to improve our ability to classify the type of cyst using a combination of novel radiomic features and cyst fluid proteolytic activity. METHODS/STUDY POPULATION: Preoperative pancreatic protocol CT images from 30 patients with proteolytic assay characterization, followed by surgical resection with a pathologically confirmed pancreatic cyst diagnosis between 2016-2019 will be used in this study. We will contour images using the widely available software 3D Slicer, and extract radiomic features using IBEX software. We will analyze area under the ROC curves to identify the radiomic features that best differentiate mucinous from non-mucinous cysts, and identify features to be cross validated. The predictive ability of identified radiomic features combined with proteolytic assay will be determined by performing multiple logistic regression analysis and comparing AUROC analysis. We will determine sensitivity and specificity for individual, as well as combinations of, analytes to determine the optimal classifier. RESULTS/ANTICIPATED RESULTS: We anticipate that the predictive ability, sensitivity, and specificity of utilizing radiomic features combined with proteolytic assay data will exceed the performance of any individual test. DISCUSSION/SIGNIFICANCE OF IMPACT: This work is designed to provide a predictive radiomic model that will enable us to better identify mucinous cysts that require further evaluation, and potentially prevent unnecessary surgery in other patients. Ultimately, we would like to improve the accuracy of noninvasive radiographic evaluation using radiomic markers. CONFLICT OF INTEREST DESCRIPTION: Dr. Charles Craik is a co-founder of Alaunus Biosciences, Inc.

